# Ancilla-Assisted Generation of Photons from Vacuum via Time-Modulation of Extracavity Qubit

**DOI:** 10.3390/e25060901

**Published:** 2023-06-06

**Authors:** Marcos V. S. de Paula, William W. T. Sinesio, Alexandre V. Dodonov

**Affiliations:** 1 Institute of Physics, University of Brasilia, Caixa Postal 04455, Brasilia 70910-900, DF, Brazil; 2 International Center of Physics, Institute of Physics, University of Brasilia, Brasilia 70910-900, DF, Brazil

**Keywords:** photon generation, dynamical Casimir effect, tripartite entangled states, cavity QED, circuit QED, dressed-states, master equation

## Abstract

We propose a scheme for the generation of photons from a vacuum via time-modulation of a quantum system indirectly coupled to the cavity field through some ancilla quantum subsystem. We consider the simplest case when the modulation is applied to an artificial two-level atom (we call ‘t-qubit’, that can be located even outside the cavity), while the ancilla is a stationary qubit coupled via the dipole interaction both to the cavity and t-qubit. We find that tripartite entangled states with a small number of photons can be generated from the system ground state under resonant modulations, even when the t-qubit is far detuned from both the ancilla and the cavity, provided its bare and modulation frequencies are properly adjusted. We attest our approximate analytic results by numeric simulations and show that photon generation from vacuum persists in the presence of common dissipation mechanisms.

## 1. Introduction

The dynamical Casimir effect (DCE) designates a plethora of phenomena characterized by the generation of photons (or quanta of some other field) from vacuum due to time-dependent variations of the geometry (dimensions) or material properties (e.g., the dielectric constant or conductivity) of some macroscopic system (see, e.g., the reviews [1,2,3,4,5]). It was initially investigated for Electromagnetic (EM) field in the presence of non-uniformly accelerating mirrors and cavities with oscillating boundaries or time-dependent material properties [6,7,8,9,10], but the concept was later extended to optomechanical systems [11,12], Bose–Einstein condensates and ultracold gases [13,14,15], polariton condensates [16], and spinor condensates [17,18]. Recently, DCE was implemented experimentally via periodical fast changes of the boundary conditions in circuit Quantum Electrodynamics architecture (circuit QED) [19,20,21,22] and Bose–Einstein condensates [23]. In addition to serving as a direct proof of the vacuum fluctuations [5], from the practical point of view DCE can be employed to generate non-classical states of light or of an ensemble of atoms [20,24,25].

The circuit QED architecture [26,27,28,29,30] is a handy platform for the implementation of DCE and its generalizations, since both the cavity’s and artificial atoms’ properties can be rapidly modulated by external bias, e.g., magnetic flux [31,32]. In particular, when the atom is directly coupled to the field via the dipole interaction (described by the Quantum Rabi Model [33]), a resonant time-modulation of the atomic transition frequency or the atom-field coupling strength can be used to generate photons and light–matter entangled states from the initial vacuum state [34,35,36,37]. In this case, one can view the atom as a microscopic constituent of the intracavity medium that shifts its effective frequency; moreover, such scheme benefits from leaving the Fock states of the cavity field time-independent (as opposed to the standard case of time-varying cavity frequency, when the annihilation operator and the Fock states depend explicitly on time [9]). These non-stationary circuit QED setups exhibit several important phenomena beside photon generation from vacuum, e.g., generation of atom-field entangled states and novel non-classical states of light [38,39], quantum simulation [40,41,42], implementation of quantum gates [43], engineering of effective interactions [44], implementation of quantum thermal engines [45,46], photon generation and atom-field effective coupling via multi-photon transitions [47,48], anti-dynamical Casimir effect (coherent annihilation of excitations due to external modulation) [49,50,51,52], photon generation by both temporal and spatial modulation in metamaterials [53], vacuum Casimir–Rabi oscillations in optomechanical systems [54], etc. [5].

In this paper, we investigate whether photons can also be generated from the vacuum by modulating an artificial atom that does not interact directly with the cavity, but instead is *indirectly coupled* to the field through some auxiliary subsystem—the *ancilla*. Such a coupling scheme may have several reasons and applications. For instance, the artificial atom can be designed specifically to withstand fast external modulation of arbitrary format, at the expense of null coupling to the cavity field but large coupling to other subsystems (possibly to different kinds of artificial atoms); or the atom can be placed outside or at the end of the cavity (at the node of the electric field) to minimize the influence of external driving on the cavity field and increase the cavity quality factor. In addition, the modulated artificial atom can be designed to couple selectively to multiple cavities by means of different stationary ancillas, which are constructed with reduced dissipative losses and enhanced atom-field coupling strengths (ultrastrong coupling [33,55], for instance). Independently of the concrete scenario, it seems timely to investigate whether such indirectly coupled time-modulated atom can be harnessed to generate photons from vacuum or engineer some useful effective interactions, and under which conditions these processes are optimized.

We address analytically and numerically this issue by considering the simplest scenario in which the time-modulated artificial atom is a qubit (“*t-qubit*”, for shortness) and the ancilla is a stationary qubit dipole-coupled to both the cavity field and the t-qubit. We find that photon generation with sufficiently large transition rates is possible provided there is a fine tuning of both the modulation frequency and the bare frequency of the t-qubit, which depend on all other system parameters.

This paper is organized as follows. The mathematical formulation of our proposal is given in Section 2, and in Section 3 we present a closed analytic description of the dynamics in terms of the system dressed-states. In Section 4, we confirm our analytic predictions by exact numeric simulations and illustrate typical system behavior in different regimes of operation. In particular, we show that the initial vacuum state can be deterministically driven either to states with only two excitations or states with multiple excitations, even in the presence of weak dissipative effects. Section 5 contains the conclusions.

## 2. Mathematical Formulation

**Figure 1:**
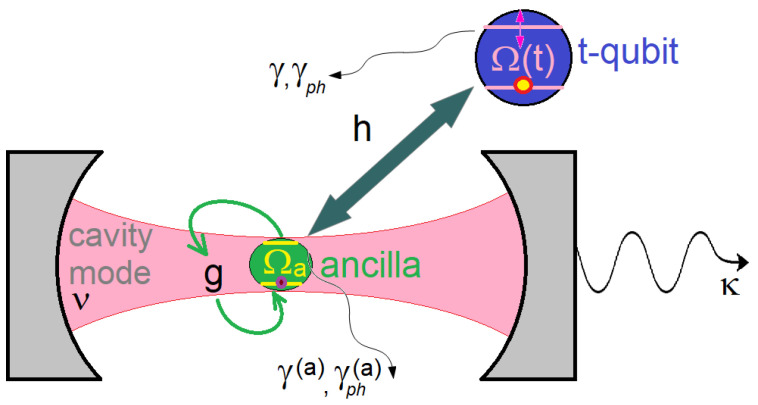
Schematic of the proposal. Extracavity *t-qubit* [of time-dependent frequency Ω(t)] is coupled to the *ancilla* (of frequency $\Omega_{a}$) with coupling strength *h*. The ancilla is coupled to the cavity mode (of frequency ν and damping rate κ) with coupling constant *g*. T-qubit (ancilla) also has damping and pure dephasing rates γ and $\gamma_{ph}$ ($\gamma^{\left(a\right)}$ and $\gamma_{ph}^{\left(a\right)}$).

Our tripartite system is represented schematically in Figure 1, and is described by the Hamiltonian (ℏ=1)

(1) $$\hat{H}=\left[\nu \hat{n}+\Omega \left(t\right){\hat{\sigma }}_{e}+h{\hat{\sigma }}_{x}{\hat{\sigma }}_{x}^{\left(a\right)}\right]+\left[\Omega_{a}{\hat{\sigma }}_{e}^{\left(a\right)}+g\left(\hat{a}+{\hat{a}}^{†}\right){\hat{\sigma }}_{x}^{\left(a\right)}\right]\;.$$

The terms in the first brackets describe the free cavity field and the t-qubit coupled to the ancilla, while the terms in the second brackets describe the ancilla qubit and its coupling to the cavity field. ν is the cavity frequency, $\hat{n}={\hat{a}}^{†}\hat{a}$ is the photon number operator and $\hat{a}$ (${\hat{a}}^{†}$) is the annihilation (creation) operator. The t-qubit operators are ${\hat{\sigma }}_{e}=\left|e\rangle \langle e\right|$, ${\hat{\sigma }}_{z}=\left|e\rangle \langle e\right|-\left|g\rangle \langle g\right|$, ${\hat{\sigma }}_{-}=\left|g\rangle \langle e\right|$, ${\hat{\sigma }}_{+}={\hat{\sigma }}_{-}^{†}$ and ${\hat{\sigma }}_{x}={\hat{\sigma }}_{+}+{\hat{\sigma }}_{-}$, where |g〉 (|e〉) denotes the ground (excited) state. For the ancilla, the operators are similar and are indicated by the upper index (a), while its ground and excited states are denoted as $|g_{a}\rangle$ and $|e_{a}\rangle$, respectively. We assume that the ancilla, with constant transition frequency $\Omega_{a}$, interacts directly with the cavity field via the dipole interaction with the time-independent coupling strength *g*. The t-qubit is not directly coupled to the cavity field; instead, it interacts with the ancilla via the dipole interaction with the coupling constant *h*. We shall derive closed (approximate) analytic description of the dynamics for moderate coupling strengths, g,h≲0.05ν, but our numeric simulations will also explore some interesting phenomena in the ultrastrong coupling regime with g,h∼ 0.1–0.3ν. Notice that the direct qubit–qubit coupling occurs naturally in many circuit QED setups [56,57,58,59], and the coupling constant *h* can be calculated in terms of the parameters of the superconducting quantum interference devices (SQUIDs) that form the qubits [60,61]. A related case in which the t-qubit is coupled simultaneously to two cavity modes was recently studied in [62], while the possibility of coupling distant qubits using a chiral ring resonator was analyzed in [63].

We assume that the transition frequency of the t-qubit is modulated externally as

(2) $$\Omega \left(t\right)=\Omega_{0}+\varepsilon \sin\left(\eta t\right)\;,$$

where $\Omega_{0}$ is the bare (average) frequency, $\varepsilon \ll \Omega_{0}$ is the modulation amplitude and η is the frequency of modulation. Notice that a periodic external modulation of the system Hamiltonian is a current topic of research in many areas of physics, e.g., two-dimensional electron systems with Rashba spin–orbit coupling irradiated by an off-resonant high-frequency electromagnetic field [64]. Moreover, the generalization of our scheme to non-harmonic modulations is straightforward using the Fourier decomposition [35], while a clever choice of the time-dependence of the modulation frequency η(t) could enhance the photon generation process and originate novel dynamical behaviors [34,65].

In circuit QED, the Hamiltonian alone does not describe accurately the dynamics due to the system coupling to the environment, so instead of the Schrödinger Equation (SE) one has to use the master equation for the density operator $\hat{\rho }$

(3) $$\frac{d\hat{\rho }}{dt}=-i\left[\hat{H},\hat{\rho }\right]+\hat{L}\hat{\rho },$$

where the Liouvillian $\hat{L}$ accounts for the influence of the environment [66,67]. The form of the Liouvillian depends on the spectral density of the reservoir and the type of coupling to the system [68]. For moderate coupling strengths one can use the standard Markovian master Equation (SMME) of quantum optics [69]

(4) $$\hat{L}={\hat{L}}_{d}+{\hat{L}}_{ph}+{\hat{L}}_{d}^{\left(a\right)}+{\hat{L}}_{ph}^{\left(a\right)}+{\hat{L}}_{\kappa },$$

where the superoperator ${\hat{L}}_{d}$ (${\hat{L}}_{ph}$) describes the energy damping (pure dephasing) of the t-qubit by a thermal reservoir. ${\hat{L}}_{d}^{\left(a\right)}$ and ${\hat{L}}_{ph}^{\left(a\right)}$ have similar meaning for the ancilla, and ${\hat{L}}_{\kappa }$ describes the cavity damping. Indeed, it was shown in [50,65] that in similar setups the discrepancy between this equation and a more rigorous one (a microscopic derivation taking into account the qubit–resonator coupling [70]) is small for the coupling strengths g≲0.1ν, while the qualitative agreement is excellent.

In this paper, we consider the zero-temperature limit of the SMME

(5) $${\hat{L}}_{d}=\gamma D\left[{\hat{\sigma }}_{-}\right]\;,\;\;{\hat{L}}_{ph}=\frac{\gamma_{ph}}{2}D\left[{\hat{\sigma }}_{z}\right]\;,\;{\hat{L}}_{\kappa }=\kappa D\left[\hat{a}\right]$$

where γ ($\gamma_{ph}$) is the atom relaxation (pure dephasing) rate, κ is the cavity damping rate and

(6) $$D\left[\hat{\Phi }\right]\hat{\rho }\equiv \frac{1}{2}\left(2\hat{\Phi }\hat{\rho }{\hat{\Phi }}^{†}-{\hat{\Phi }}^{†}\hat{\Phi }\hat{\rho }-\hat{\rho }{\hat{\Phi }}^{†}\hat{\Phi }\right)$$

is the so called Lindblad superoperator that preserves the hermiticity, normalization and positivity of $\hat{\rho }$ [68]. For numeric simulations in Section 4 we shall adopt the following parameters:

(7) $$\gamma^{\left(a\right)}=5\times 10^{-5}\nu \;,\;\gamma_{ph}^{\left(a\right)}=\kappa =\frac{\gamma^{\left(a\right)}}{2}\;,\;\gamma =5\gamma^{\left(a\right)}\;,\;\gamma_{ph}=\frac{\gamma }{2}\;.$$

This represents the presumable situation in which the t-qubit is exposed to moderate dissipation due to external modulation, while the ancilla is less susceptible to dissipation by proper design. These dissipative rates were assumed sufficiently small yet readily achievable experimentally [71,72,73]. Such approximate treatment is sufficient to assess the feasibility of our scheme in realistic situations, and the parameters in Equation (7) can be viewed as a benchmark for sufficient dissipation rates. We note that an accurate description of the dissipative dynamics would require solving the microscopic master Equation [70], for which the spectral densities of the baths must be known. Moreover, the driving field could cause the system heating. Since in our scheme the t-qubit is placed outside or at the end of the cavity, such undesirable effects are minimized and could be taken into account by the additional dissipative term $\gamma_{heat}D\left[{\hat{\sigma }}_{+}\right]$ in Equation (4).

## 3. Analytic Description

The analysis is simplified by introducing a new “conjoint” atomic basis $\left\{|A_{i}\rangle ,i=0,\ldots ,3\right\}$, in which $|A_{i}\rangle$ are the eigenstates of the two-atom time-independent Hamiltonian ${\hat{H}}_{a}=\Omega_{0}{\hat{\sigma }}_{e}+\Omega_{a}{\hat{\sigma }}_{e}^{\left(a\right)}+h{\hat{\sigma }}_{x}{\hat{\sigma }}_{x}^{\left(a\right)}$ containing the bare t-qubit frequency $\Omega_{0}$:

(8) $$\begin{array}{ccc} |A_{0}\rangle & = & N_{0}\left[\left(W_{+}+D_{+}\right)|g,g_{a}\rangle -h|e,e_{a}\rangle \right]\\ |A_{1}\rangle & = & N_{1}\left[\left(W_{-}-D_{-}\right)|g,e_{a}\rangle +h|e,g_{a}\rangle \right]\\ |A_{2}\rangle & = & N_{2}\left[\left(W_{-}+D_{-}\right)|g,e_{a}\rangle +h|e,g_{a}\rangle \right]\\ |A_{3}\rangle & = & N_{3}\left[\left(W_{+}-D_{+}\right)|g,g_{a}\rangle -h|e,e_{a}\rangle \right]\;. \end{array}$$

Here $W_{\pm }=\left(\Omega_{a}\pm \Omega_{0}\right)/2$, $D_{\pm }=\sqrt{W_{\pm }^{2}+h^{2}}$ and $N_{i}$ are the normalization constants (with the dimension 1/frequency); some useful approximate formulae for the eigenstates $|A_{i}\rangle$ are given in the Appendix A. The corresponding eigenenergies are

(9) $$\begin{array}{ccc} \lambda_{0} & = & W_{+}-D_{+}\;,\;\lambda_{1}=W_{+}-D_{-}\\ \lambda_{2} & = & W_{+}+D_{-}\;,\;\lambda_{3}=W_{+}+D_{+}\;. \end{array}$$

In the basis $\left\{|A_{i}\rangle \right\}$ the time-independent system Hamiltonian reads

(10) $${\hat{H}}_{0}=\nu \hat{n}+\sum_{i=0}^{3}\lambda_{i}|A_{i}\rangle \langle A_{i}|+g\left(\hat{a}+{\hat{a}}^{†}\right){\hat{\sigma }}_{x}^{\left(a\right)}\;$$

and the total Hamiltonian is $\hat{H}={\hat{H}}_{0}+\varepsilon \sin\left(\eta t\right){\hat{\sigma }}_{e}$.

### 3.1. Spectrum Far from Degeneracies

Far from any degeneracy between the eigenenergies of ${\hat{H}}_{0}\left(g=0\right)$ (system Hamiltonian without the matter–field coupling) the spectrum of ${\hat{H}}_{0}$ can be found from the non-degenerate perturbation theory. We use the orthonormal complete basis $|A_{i}^{n}\rangle \equiv |A_{i}\rangle ⊗|n\rangle$ (where |n〉 is the Fock state of the field with n≥0 and i=0,…,3) and denote the eigenstate (eigenvalue) of ${\hat{H}}_{0}$ with the *dominant contribution* of the state $|A_{i}^{n}\rangle$ as $|A_{i},n\rangle$ ($\lambda_{i,n}$). For example, to the second order in *g* one obtains the (non-normalized) state

(11) $$\begin{array}{ccc} & & |A_{0},n\rangle =|A_{0}^{n}\rangle +g\left[\frac{\sigma_{01}\sqrt{n}}{v-D_{+}+D_{-}}|A_{1}^{n-1}\rangle -\frac{\sigma_{01}\sqrt{n+1}}{v+D_{+}-D_{-}}|A_{1}^{n+1}\rangle \right.\\ & & \left.+\frac{\sigma_{02}\sqrt{n}}{v-D_{+}-D_{-}}|A_{2}^{n-1}\rangle -\frac{\sigma_{02}\sqrt{n+1}}{v+D_{+}+D_{-}}|A_{2}^{n+1}\rangle \right]\\ & & +g^{2}\left[\sqrt{n(n-1)}\left(\Xi_{n}^{\left(1\right)}|A_{0}^{n-2}\rangle +\Xi_{n}^{\left(4\right)}|A_{3}^{n-2}\rangle \right)\right.\\ & & \left.+\sqrt{\left(n+1)(n+2\right)}\left(\Xi_{n}^{\left(2\right)}|A_{0}^{n+2}\rangle +\Xi_{n}^{\left(5\right)}|A_{3}^{n+2}\rangle \right)+\Xi_{n}^{\left(3\right)}|A_{3}^{n}\rangle \right]\;, \end{array}$$

where $\sigma_{ij}\equiv \langle A_{i}|{\hat{\sigma }}_{x}^{\left(a\right)}|A_{j}\rangle$ and the coefficients $\Xi_{n}^{\left(k\right)}$ are given in the Appendix A. The corresponding eigenenergy reads

(12) $$\begin{array}{ccc} \lambda_{0,n} & = & \lambda_{0}+vn+g^{2}\left[\frac{\sigma_{01}^{2}n}{v-D_{+}+D_{-}}-\frac{\sigma_{01}^{2}\left(n+1\right)}{v+D_{+}-D_{-}}\right.\\ & & +\left.\frac{\sigma_{02}^{2}n}{v-D_{+}-D_{-}}-\frac{\sigma_{02}^{2}\left(n+1\right)}{v+D_{+}+D_{-}}\right]\;. \end{array}$$

Expressions for other states $|A_{i},n\rangle$ and eigenenergies $\lambda_{i,n}$ can be obtained similarly, but they are not required for the present work.

### 3.2. Spectrum near Degeneracies

Most accessible applications of our scheme explore the regime of parameters in which two states of the subspace $A_{n}=\{|A_{0}^{n}\rangle$,$|A_{1}^{n-1}\rangle$, $|A_{2}^{n-1}\rangle$, $|A_{3}^{n-2}\rangle \}$ are nearly degenerate, as occurs for $D_{+}\pm D_{-}\approx \nu$ (when $\Omega_{0}\approx \nu$ or $\Omega_{a}\approx \nu$) or $\Omega_{a}+\Omega_{0}\approx 2\sqrt{\nu^{2}-h^{2}}$. In this case, the non-degenerate perturbation theory fails, but one can obtain excellent analytic results by expanding ${\hat{H}}_{0}$ in the subspace $A_{n}$ as

(13) $$\Upsilon_{n}=X\;\hat{I}+M_{n}\;,$$

where $X=\nu n+\lambda_{0}$, $\hat{I}$ is the 4 × 4 identity matrix and

(14) $$M_{n}=\left(\begin{array}{cccc} 0 & a & b & 0\\ a & x & 0 & -c\\ b & 0 & y & d\\ 0 & -c & d & z \end{array}\right)$$

(the parameters of $M_{n}$ are given in the Appendix A). The eigenvalues $\Lambda_{n,i}$ of $M_{n}$ (with i=1,…,4 for a given *n*, where *n* labels the subspace $A_{n}$) can be found exactly using the Ferrari’s method. The normalized eigenstate corresponding to the eigenvalue $\Lambda_{n,i}$ is

(15) $$|\phi_{n,i}\rangle =\phi_{n,i}^{\left(0\right)}|A_{0}^{n}\rangle +\phi_{n,i}^{\left(1\right)}|A_{1}^{n-1}\rangle +\phi_{n,i}^{\left(2\right)}|A_{2}^{n-1}\rangle +\phi_{n,i}^{\left(3\right)}|A_{3}^{n-2}\rangle \;,$$

where $\phi_{n,i}^{\left(k\right)}$ are the probability amplitudes of the conjoint atomic state $|A_{k}\rangle$ (see the Appendix A for the derivation). The eigenenergy of the state $|\phi_{n,i}\rangle$ is denoted as $\lambda_{n,i}^{\phi }$ and reads $\lambda_{n,i}^{\phi }=X+\Lambda_{n,i}$. For example, according to our notation, near the degeneracy the eigenstate $|A_{0},n\rangle$ of $\Upsilon_{n}$ is the state $|\phi_{n,i=J}\rangle$ for which $\phi_{n,i=J}^{\left(0\right)}$ is the largest among the four states $\left\{|\phi_{n,i}\rangle \right\}$; the eigenenergy $\lambda_{0,n}$ is the corresponding function $\lambda_{n,i=J}^{\phi }$ (so $\lambda_{0,n}$ can be a discontinuous function of parameters near the degeneracy).

The above diagonalization procedure has one drawback, it neglects the coupling of the subspace $A_{n}$ to the neighboring subspaces $A_{n\pm 2}$, carried by the counter-rotating terms $g(\hat{a}{\hat{\sigma }}_{-}^{\left(a\right)}+h.c.)$ and $h({\hat{\sigma }}_{-}{\hat{\sigma }}_{-}^{\left(a\right)}+h.c.)$ in the Hamiltonian ${\hat{H}}_{0}$ (notice that the counter-rotating terms were fully taken into account within the subspace $A_{n}$). For small values of *g* the main effect of the neglected contributions is to introduce small frequency shifts (“Bloch–Siegert” shifts [35]) to the eigenfrequencies of the bare Hamiltonian ${\hat{H}}_{0}\left(g=0\right)$. To include these corrections in a simplified manner, we consider the subspace $B_{n}=\{|A_{0}^{n-2}\rangle$,$|A_{1}^{n-1}\rangle$, $|A_{2}^{n-1}\rangle$,$|A_{3}^{n}\rangle \}$ containing the basis states connected solely by the counter-rotating terms. In this basis, the 4 × 4 matrix corresponding to the Hamiltonian ${\hat{H}}_{0}$ is ${\tilde{\Upsilon }}_{n}=\left[\nu \left(n-2\right)+\lambda_{0}\right]\hat{I}+{\tilde{M}}_{n}$, where

(16) $${\tilde{M}}_{n}=\left(\begin{array}{cccc} 0 & g\sqrt{n-1}\sigma_{01} & g\sqrt{n-1}\sigma_{02} & 0\\ g\sqrt{n-1}\sigma_{01} & \nu_{D}-D_{-} & 0 & -g\sqrt{n}\sigma_{02}\\ g\sqrt{n-1}\sigma_{02} & 0 & \nu_{D}+D_{-} & g\sqrt{n}\sigma_{01}\\ 0 & -g\sqrt{n}\sigma_{02} & g\sqrt{n}\sigma_{01} & 2\nu_{D} \end{array}\right)$$

has the same structure as $M_{n}$ (with $\nu_{D}\equiv \nu +D_{+}$), so its eigenvalues can be found as previously. Denoting the eigenvalues of ${\tilde{M}}_{n}$ in the increasing order as ${\tilde{\Lambda }}_{n,i}$ (i=1,…,4), the frequency shifts of the states in the subspace $B_{n}$ are found as $\delta_{0}^{n-2}\equiv {\tilde{\Lambda }}_{n,1}$, $\delta_{1}^{n-1}\equiv {\tilde{\Lambda }}_{n,2}-\left(\nu +D_{+}-D_{-}\right)$, $\delta_{2}^{n-1}\equiv {\tilde{\Lambda }}_{n,3}-\left(\nu +D_{+}+D_{-}\right)$, and $\delta_{3}^{n}\equiv {\tilde{\Lambda }}_{n,4}-2\left(\nu +D_{+}\right)$ ($\delta_{l}^{k}$ is the frequency shift of the state $|A_{l}^{k}\rangle$). Now one can insert these shifts into the matrix (13) by replacing $X\to X+\delta_{0}^{n}$, $x\to x+\delta_{1}^{n-1}-\delta_{0}^{n}$, $y\to y+\delta_{2}^{n-1}-\delta_{0}^{n}$, and $z\to z+\delta_{3}^{n-2}-\delta_{0}^{n}$, obtaining analytically the eigenvalues and eigenstates of the Hamiltonian ${\hat{H}}_{0}$ near the degeneracy points. As will be shown in Section 4, for moderate coupling strengths g≲0.05ν, this procedure is in excellent agreement with exact numeric results.

For higher values of *g* or direct transitions involving n>2 photons, the above diagonalization procedure becomes improper (since $A_{n}$ only contains states differing by at most two photons) and must be generalized by adding more states to the subspace $A_{n}$ (forming a larger subspace $A_{n}^{gen}$). However, since there is no algebraic solution to general polynomial equations of degree five or higher with arbitrary coefficients (Abel–Ruffini theorem), it is easier to perform the diagonalization numerically, as will be completed in Section 4.1.

### 3.3. Dynamics in the Dressed-Basis

The unitary system dynamics is straightforward in terms of the eigenstates (*dressed-states*) of ${\hat{H}}_{0}$, which can be found either numerically or analytically as in the previous subsections. Expanding the wavefunction as

(17) $$|\psi \left(t\right)\rangle =\sum_{l=0}^{\infty }e^{-itE_{l}}A_{l}\left(t\right)|\varphi_{l}\rangle \;,$$

where $|\varphi_{l}\rangle$ and $E_{l}$ are the eigenstates and eigenvalues of ${\hat{H}}_{0}$ in the increasing order ($E_{l+1}\geq E_{l}$), we obtain for the probability amplitudes

(18) $$i{\dot{A}}_{m}=\varepsilon \sin\left(\eta t\right)\sum_{l=0}^{\infty }A_{l}e^{-itE_{lm}}\langle \varphi_{m}|{\hat{\sigma }}_{e}|\varphi_{l}\rangle \;,$$

where $E_{lm}=E_{l}-E_{m}$ is the energy difference. For the realistic case of weak modulations, ε≪η, one can neglect the rapidly oscillating terms to obtain

(19) $${\dot{A}}_{m}=-\sum_{l\neq m}^{\infty }\mathrm{sign}\left(E_{lm}\right)e^{-\mathrm{sign}\left(E_{lm}\right)it\left(\left|E_{lm}\right|-\eta \right)}R_{m;l}A_{l}\;,$$

where

(20) $$R_{m;l}\equiv \frac{\varepsilon }{2}\langle \varphi_{m}|{\hat{\sigma }}_{e}|\varphi_{l}\rangle \;.$$

Therefore, for the resonant modulation frequency $\eta =\left|E_{lm}\right|$ the external perturbation induces transition between the dressed-states $|\varphi_{m}\rangle$ and $|\varphi_{l}\rangle$ with the transition rate $|R_{m;l}|$. From the practical standpoint, the numeric results are obtained by finding the eigenvalues $E_{m}$ and eigenstates $|\varphi_{m}\rangle$ of ${\hat{H}}_{0}$ in the basis $\left\{|A_{k}^{n}\rangle \right\}$, where k=0,…,3 and $n=0,\ldots ,n_{tr}$, and then evaluating $R_{m;l}$ and $E_{lm}$. $n_{tr}$ stands for the maximum number of photons fixed by the truncation procedure, which does not affect the results for eigenstates with photon numbers $n\ll n_{tr}$ (here the value $n_{tr}=30$ was enough). For analytic calculations, one simply uses the closed form expressions for dressed-states and eigenenergies found in Section 3.1 (for the states $|A_{i},n\rangle$ far from degeneracy points, with eigenenergies $\lambda_{i,n}$) and Section 3.2 (for dressed-states $|\phi_{n,i}\rangle$ near degeneracy points, with eigenenergies $\lambda_{n,i}^{\phi }$) to evaluate the transition rate and energy differences. Since all the states were expanded in the common basis $\left\{|A_{k}^{n}\rangle \right\}$, such calculations are long but straightforward.

In this work, we are primarily interested in photon generation from the initial (non-degenerate) ground state of the system $|A_{0},0\rangle$. Near the degeneracy points one can obtain approximate analytic expression for the transition rate using the formulae (11) and (15). To the first order in *g*, the transition rate to the two-excitations state $|\phi_{2,i}\rangle$ of subspace $A_{2}$ is

(21) $$R_{0,0;2,i}=\frac{\varepsilon h^{2}}{2}\left[N_{0}N_{3}\phi_{2,i}^{\left(3\right)}-gT_{i}\right]\;,$$

where

(22) $$T_{i}=\left(\frac{\sigma_{01}N_{1}}{v+D_{+}-D_{-}}+\frac{\sigma_{02}N_{2}}{v+D_{+}+D_{-}}\right)\left(N_{1}\phi_{2,i}^{\left(1\right)}+N_{2}\phi_{2,i}^{\left(2\right)}\right)\;.$$

Analogously, the transition rate between the dressed-states $|\phi_{n,i}\rangle$ and $|\phi_{n+2,j}\rangle$ is

(23) $$R_{n,i;n+2,j}=\frac{\varepsilon }{2}\langle \phi_{n,i}|{\hat{\sigma }}_{e}|\phi_{n+2,j}\rangle =\frac{\varepsilon }{2}N_{0}N_{3}h^{2}\phi_{n,i}^{\left(0\right)}\phi_{n+2,j}^{\left(3\right)}\;.$$

**Figure 2:**
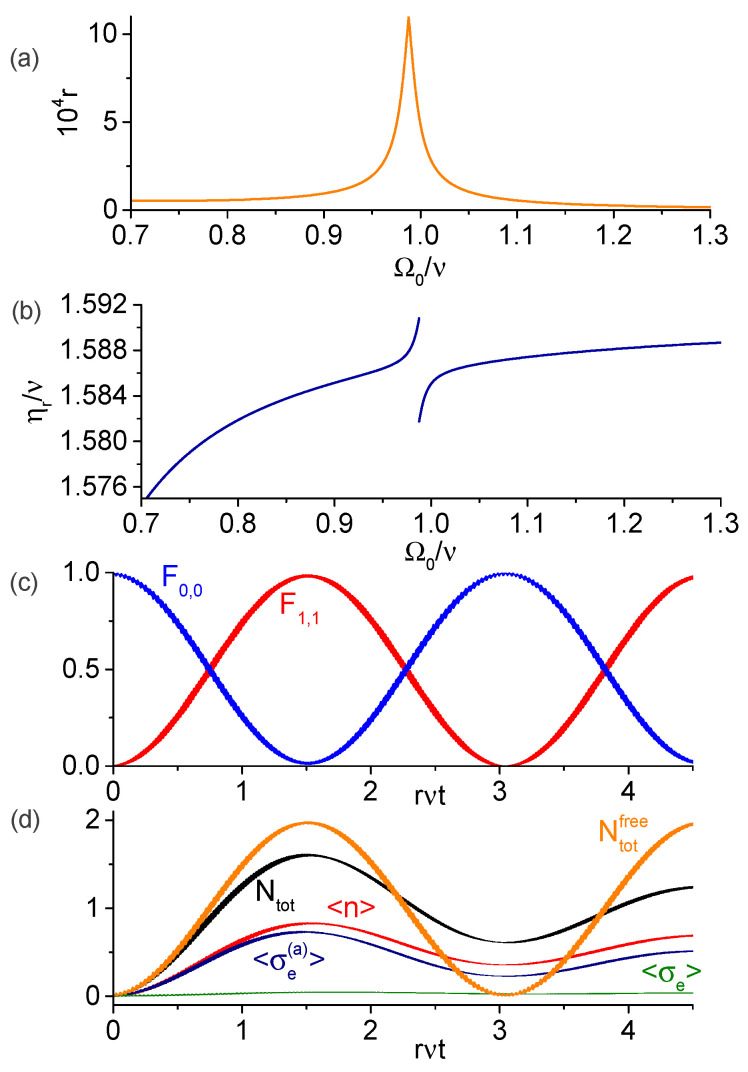
Numeric results for the transition $|A_{0},0\rangle ↔|A_{1},1\rangle$ using dispersive ancilla with frequency $\Omega_{a}=0.6\nu$. (**a**) Dimensionless transition rate $r=|R_{0,0;1,1}|/\nu$ as function of $\Omega_{0}/\nu$. (**b**) Resonant modulation frequency, in which the discontinuity arises because the state $|A_{1},1\rangle$ is different below and above the degeneracy between the bare states $|A_{0}^{2}\rangle$ and $|A_{2}^{1}\rangle$. (**c**) Fidelities of the states $|A_{0},0\rangle$ and $|A_{1},1\rangle$ obtained via numeric solution of the SE with the total Hamiltonian (1). As expected, the period of oscillation is $\pi {\left(\nu r\right)}^{-1}$. (**d**) Dynamics of the average photon number 〈n〉, qubits excitations $\langle \sigma_{e}\rangle$ and $\langle \sigma_{e}^{\left(a\right)}\rangle$, and the total number of excitations $N_{tot}$ obtained via numeric solution of the master equation in the presence of dissipation. For comparison, $N_{tot}^{free}$ illustrates the average total number of excitations without dissipation, when it attains the maximum value $N_{tot}^{free}=2$.

**Figure 3:**
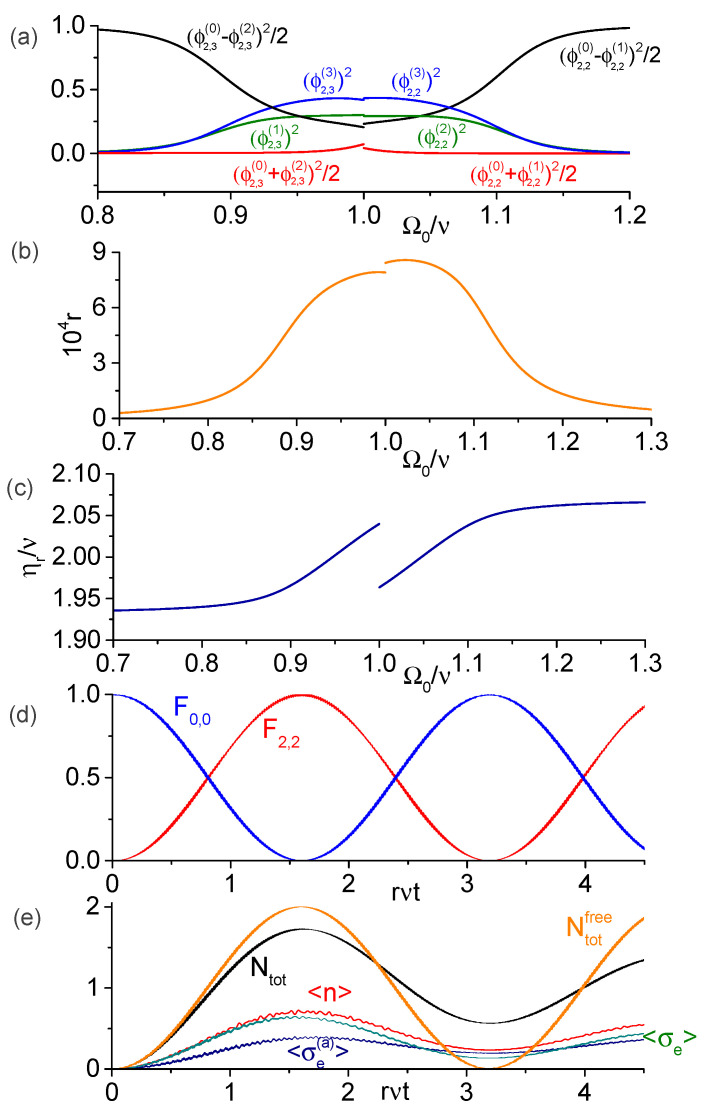
Transition from $|A_{0},0\rangle$ to the two-excitations states $|\phi_{2,i}\rangle$ (i=3 for $\Omega_{0}<\nu$, i=2 for $\Omega_{0}>\nu$) for resonant ancilla with frequency $\Omega_{a}=\nu$. (**a**) Composition of the dressed-states (15) involved in the studied transition. (**b**) Dimensionless transition rate $r=|R_{0,0;\phi_{2},i}|/\nu$ as function of t-qubit’s bare frequency. (**c**) Resonant modulation frequency. (**d**) Fidelities of the states $|A_{0},0\rangle$ and $|\phi_{2,2}\rangle$ obtained from numeric solution of the SE for $\Omega_{a}=\nu$ and $\Omega_{0}=1.05\nu$. (**e**) Numeric dynamics of the average numbers of excitations ($\langle n\rangle ,\langle \sigma_{e}\rangle ,\langle \sigma_{e}^{\left(a\right)}\rangle ,N_{tot}$) in the presence of dissipation, compared to the average total number of excitations $N_{tot}^{free}$ under unitary evolution.

**Figure 4:**
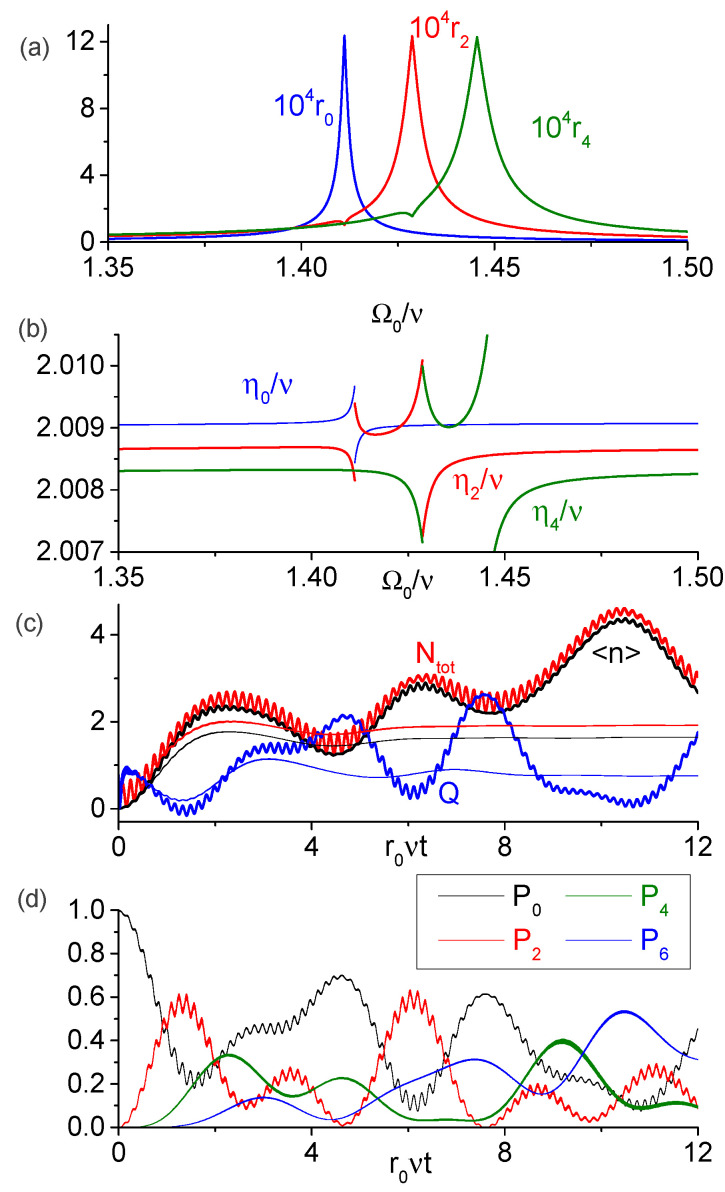
DCE-like transition in which $|A_{0},n\rangle$ couples to the state $|A_{0},n+2\rangle$ with even *n* for off-resonant ancilla with $\Omega_{a}=0.6\nu$. (**a**) Dimensionless transition rates $r_{n}=\left|R_{0,n;0,n+2}\right|/\nu$. (**b**) Resonant modulation frequencies, where discontinuities occur due to the modification of the dressed-states at the degeneracy points. (**c**) Numeric dynamics of the average photon number, total number of excitations and the Mandel’s Q-factor. Bold (thin) lines denote the unitary (dissipative) evolution. Notice that even with dissipation the generation of several excitations is possible. (**d**) Dynamics of the cavity Fock states with populations above 10% during unitary evolution. In this example, up to six photons can be generated with substantial probabilities, while the probability of the vacuum state can decrease below 10%.

**Figure 5:**
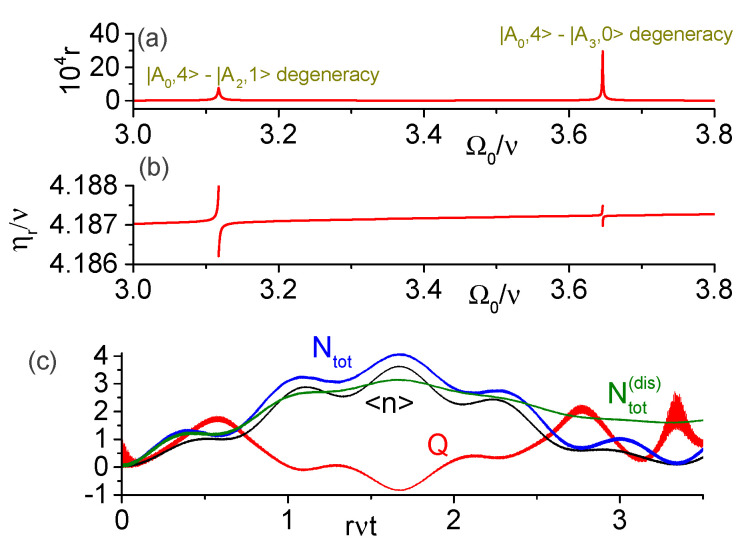

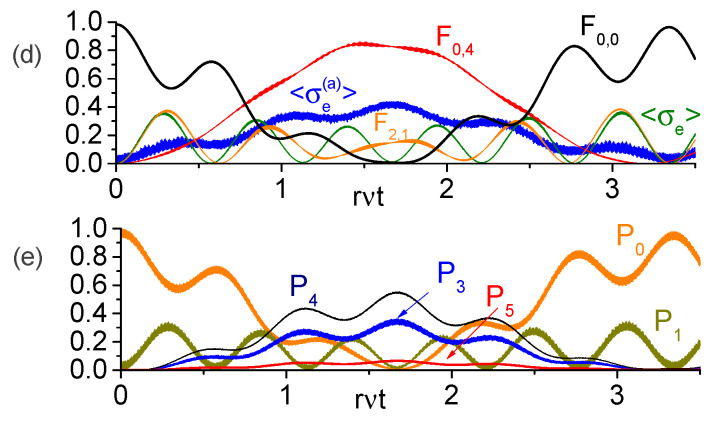
Numeric results for the direct transition $|A_{0},0\rangle \to |A_{0},4\rangle$ in the ultrastrong coupling regime for parameters $\Omega_{a}=0.6\nu$, g=0.2ν, h=0.1ν. (**a**) Dimensionless transition rate $r=|R_{0,0;0,4}|/\nu$; the peaks occur near degeneracies between the states $\{|A_{0},4\rangle ,|A_{2},1\rangle \}$ and $\{|A_{0},4\rangle ,|A_{3},0\rangle \}$. (**b**) Resonant modulation frequency $\eta_{r}=E_{0,4}-E_{0,0}$. (**c**) Average photon number, average total number of excitations, and the Mandel’s Q-factor for $\Omega_{0}=3.12\nu$ and η=4.1873ν under unitary evolution. $N_{tot}^{\left(dis\right)}$ is the total excitation number according to SMME (it is the only quantity displayed under dissipation). (**d**) Populations of t-qubit and ancilla; fidelities of the states $|A_{0},0\rangle$, $|A_{0},4\rangle$ and $|A_{2},1\rangle$. (**e**) Dynamics of the Fock states with occupation probabilities above 5%.

**Figure 6:**
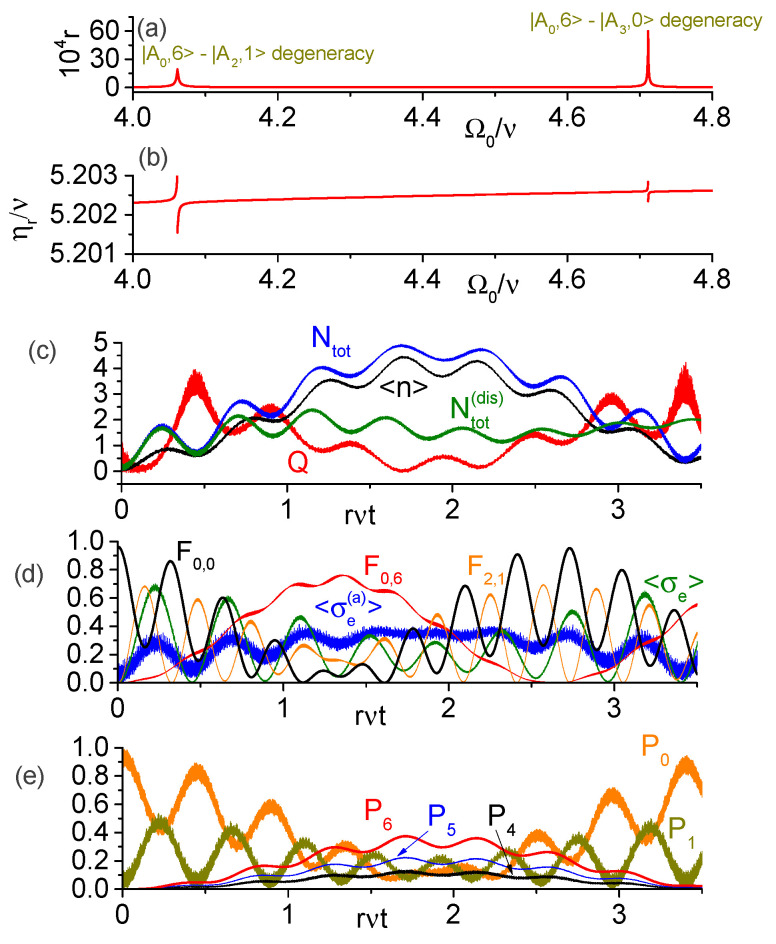
Numeric results for the direct transition $|A_{0},0\rangle \to |A_{0},6\rangle$ in the ultrastrong coupling regime for parameters $\Omega_{a}=0.6\nu$, g=0.3ν, h=0.2ν. (**a**) Dimensionless transition rate $r=|R_{0,0;0,6}|/\nu$; the peaks occur near degeneracies between the states $\{|A_{0},6\rangle ,|A_{2},1\rangle \}$ and $\{|A_{0},6\rangle ,|A_{3},0\rangle \}$. (**b**) Resonant modulation frequency $\eta_{r}=E_{0,6}-E_{0,0}$. (**c**) Average photon number, average total number of excitations, and the Mandel’s Q-factor for $\Omega_{0}=4.057\nu$ and η=5.201ν under unitary evolution, as well as $N_{tot}^{\left(dis\right)}$ according to the SMME. (**d**) Populations of t-qubit and ancilla; fidelities of the states $|A_{0},6\rangle$, $|A_{2},1\rangle$, and $|A_{0},0\rangle$. (**e**) Dynamics of the Fock states with occupation probabilities above 10%.

When the modulation frequency matches only one possible value $\left|E_{lm}\right|$ (with non-zero transition rate $R_{m;l}$ between the respective eigenstates), the dressed-states $|\varphi_{m}\rangle$ and $|\varphi_{l}\rangle$ become resonantly coupled and exhibit sinusoidal behaviors (see Figure 2 and Figure 3 below), ${\left|A_{m}\left(t\right)\right|}^{2}=\cos^{2}\left(R_{m;l}t\right)$ and ${\left|A_{l}\left(t\right)\right|}^{2}=\sin^{2}\left(R_{m;l}t\right)$ (assuming that only $A_{m}$ was initially non-zero). On the other hand, when several values $\left|E_{lm}\right|$ are close to the modulation frequency (with the mismatches $\eta -\left|E_{lm}\right|$ smaller or of the order of $|R_{m;l}|$), then several dressed-states can become simultaneously coupled and the dynamics becomes more intricate (see Figure 4, Figure 5 and Figure 6). We also note that the neglected rapidly oscillating terms introduce small corrections to the resonant frequencies $|E_{lm}|$ [35,49], which are found numerically in this paper.

The ground state can also be directly coupled to dressed-states with more than two excitations. To obtain reliable analytic formulae for the resonant modulation frequencies and transition rates, one would need to generalize the results of Section 3.2 for larger subspaces (more than four states in $A_{n}^{gen}$). However, we can assure that these transitions do take place by substituting the formulae (11) and (15) into Equation (20) to obtain the (overly underestimated) 4-excitations transition rate

(24) $$R_{0,0;4,i}=\frac{\varepsilon }{2}\langle A_{0},0|{\hat{\sigma }}_{e}|\varphi_{4,i}\rangle \approx \sqrt{2!}\frac{\varepsilon }{2}h^{2}g^{2}\phi_{4,i}^{\left(3\right)}N_{3}\left(N_{0}\Xi_{0}^{\left(2\right)}+N_{3}\Xi_{0}^{\left(5\right)}\right)$$

corresponding to the transition $|A_{0},0\rangle \to |\phi_{4,i}\rangle$ of the subspace $A_{4}$. Similarly, if one expanded the ground state to the fourth order in *g* using non-degenerate perturbation theory in Equation (11), one would obtain $R_{0,0;6,i}\propto \sqrt{4!}\frac{\varepsilon }{2}h^{2}g^{4}\phi_{6,i}^{\left(3\right)}$ for the transition $|A_{0},0\rangle \to |\phi_{6,i}\rangle$ of the subspace $A_{6}$. In Section 4.1, we shall calculate the transition rates for the transition $|A_{0},0\rangle \to |A_{0},n\rangle$ with n=4 and 6 by exact numeric diagonalization of the Hamiltonian ${\hat{H}}_{0}$, showing that these transition rates are strongly enhanced in the vicinity of degeneracy between $|A_{0},n\rangle$ and the states $|A_{2},1\rangle$ or $|A_{3},0\rangle$. Since such multi-photon transitions are weaker than two-photons ones, we shall study their implementation in the ultrastrong coupling regime [33,55] with g∼ 0.2–0.3ν.

Our scheme can be readily generalized to simultaneous modulation of other system parameters, one merely needs to add the corresponding matrix element on the RHS of Equation (18), which would produce an additive contribution to the transition rate (20). The inclusion of terms proportional to $\varepsilon^{2}$ in Equation (19) is also possible [74], but is not considered here because the resulting transition rates are roughly η/ε times smaller than Equation (20) (although in this case one benefits from halved resonant modulation frequencies).

## 4. Numeric Results and Discussion

To assess the feasibility of our scheme for photon generation from vacuum in circuit QED, we first assume conservative experimental parameters g=h=0.05ν. In the following, we set the modulation amplitude as $\varepsilon =0.1\Omega_{0}$.

In Figure 2a, we plot the dimensionless transition rate $r\equiv |R_{0,0;1,1}|/\nu$ for transition from the system ground state $|A_{0},0\rangle \approx |g,g_{a},0\rangle$ to the state $|A_{1},1\rangle \approx -|g,e_{a},1\rangle$ as function of the t-qubit’s bare frequency $\Omega_{0}$ for the ancilla frequency $\Omega_{a}=0.6\nu$. Figure 2b shows the resonant modulation frequency $\eta_{r}=E_{1,1}-E_{0,0}$ (*r* and $\eta_{r}$ were obtained by diagonalizing numerically ${\hat{H}}_{0}$). This transition can be roughly interpreted as the Anti-Jaynes–Cummings ancilla–field regime in which one photon and one ancilla excitation are created, while the t-qubit remains approximately in the ground state. We verified that the relative errors between the exact numeric results and the analytic expressions of Section 3 is below 0.6% for the modulation frequency $\eta_{r}$ and below 5% for the transition rate *r* (data not shown). In Figure 2c, we show the fidelities $F_{k,n}\left(t\right)={\left|\langle A_{k},n|\psi \left(t\right)\rangle \right|}^{2}$ of the dressed-states as function of time for (k=0,n=0) and (k=1,n=1), obtained by solving numerically the SE for the original Hamiltonian (1) with parameters $\Omega_{0}=0.95\nu$, η=1.586ν, and the initial state $|g,g_{a},0\rangle$. For these parameters, the weights in the state $|A_{1},1\rangle$ [Equation (15) with properly chosen *i*] are $\phi_{2,i}^{\left(0\right)}=-0.168$, $\phi_{2,i}^{\left(1\right)}=-0.977$, $\phi_{2,i}^{\left(2\right)}=0.021$ and $\phi_{2,i}^{\left(3\right)}=0.132$ (so the contribution of the bare state $|A_{1}^{1}\rangle$ is the largest) and the transition rate is $r=1.93\times 10^{-4}$. As predicted in Section 3, there is a periodic population exchange between the dressed-states $|A_{0},0\rangle$ and $|A_{1},1\rangle$ with period π/(νr), while other states remain practically unpopulated. In Figure 2d, we present the numeric solution of the master Equation (3). We plot the average photon number $\langle \hat{n}\rangle$, the qubits excitations $\langle {\hat{\sigma }}_{e}\rangle$ and $\langle {\hat{\sigma }}_{e}^{\left(a\right)}\rangle$ and the average total number of excitations $N_{tot}=\langle \left(\hat{n}+{\hat{\sigma }}_{e}+{\hat{\sigma }}_{e}^{\left(a\right)}\right)\rangle$. For comparison, the average total number of excitation during unitary evolution (labeled $N_{tot}^{free}$) is also shown. This figure attests that photon generation from vacuum persists in the presence of dissipation. The order of magnitude of maximal allowed dissipation rates (denoted as $\gamma_{\max}$ for the atoms and $\kappa_{\max}$ for the cavity dampings) can be estimated from the condition $\gamma_{\max}/\nu ,\kappa_{\max}n_{\max}/\nu ∼r$ (where $|n_{\max}\rangle$ is highest Fock state significantly populated during the evolution, equal to $n_{\max}=1$ in this example), which for r∼$10^{-4}\nu$ are ubiquitous in several circuit QED setups [71,72,73].

In Figure 3, we consider the ancilla at the exact resonance with the cavity mode, $\Omega_{a}=\nu$, and study the transition from $|A_{0},0\rangle$ to the state $|\phi_{n=2,i}\rangle$ given by Equation (15), in which i=3 for $\Omega_{0}<\nu$ and i=2 for $\Omega_{0}>\nu$ (recall that the index *n* specifies the subspace $A_{n}$). Table 1 and Table 2 show the values of the probability amplitudes $\phi_{2,i}^{\left(j\right)}$ (j=0,⋯,3) for several values of $\Omega_{0}/\nu$ for parameters $\Omega_{a}=\nu$, g=h=0.05ν. Figure 3a shows the quantities ${\left(\phi_{2,3}^{\left(0\right)}+\phi_{2,3}^{\left(2\right)}\right)}^{2}/2$, ${\left(\phi_{2,3}^{\left(0\right)}-\phi_{2,3}^{\left(2\right)}\right)}^{2}/2$, ${\left(\phi_{2,3}^{\left(1\right)}\right)}^{2}$ and ${\left(\phi_{2,3}^{\left(3\right)}\right)}^{2}$ for $\Omega_{0}<\nu$ and ${\left(\phi_{2,2}^{\left(0\right)}+\phi_{2,2}^{\left(1\right)}\right)}^{2}/2$, ${\left(\phi_{2,2}^{\left(0\right)}-\phi_{2,2}^{\left(1\right)}\right)}^{2}/2$, ${\left(\phi_{2,2}^{\left(2\right)}\right)}^{2}$, and ${\left(\phi_{2,2}^{\left(3\right)}\right)}^{2}$ for $\Omega_{0}>\nu$. From Equation (8) we see that far from the resonance, $|\Omega_{0}-\nu |≳0.2\nu$, the system eigenstates are approximately $|\phi_{2,3}\rangle \approx |g\rangle ⊗(|g_{a},2\rangle -|e_{a},1\rangle )/\sqrt{2}$ for $\Omega_{0}<\nu$ and $|\phi_{2,2}\rangle \approx -|g\rangle ⊗(|g_{a},2\rangle +|e_{a},1\rangle )/\sqrt{2}$ for $\Omega_{0}>\nu$. Figure 3b,c show the numeric results for the dimensionless transition rate $r=|R_{0,0;\phi_{2},i}|/\nu$ (i=3 for $\Omega_{0}<\nu$ and i=2 for $\Omega_{0}>\nu$) and resonant modulation frequency $\eta_{r}=E_{2,i}^{\phi }-E_{0,0}$ (where $E_{2,i}^{\phi }$ is the numeric eigenvalue of ${\hat{H}}_{0}$ corresponding $\lambda_{2,i}^{\phi }$). The agreement with analytic results of Section 3 is excellent, with the relative error below 3% for *r* and below 0.1% for $\eta_{r}$ (data not shown). Figure 3d shows the numeric solution of the SE for the fidelities $F_{0,0}\left(t\right)={\left|\langle A_{0},0|\psi \left(t\right)\rangle \right|}^{2}$ and $F_{2,2}\left(t\right)={\left|\langle \phi_{2,2}|\psi \left(t\right)\rangle \right|}^{2}$ for parameters $\Omega_{a}=\nu$, $\Omega_{0}=1.05\nu$, and η=2.002ν, when the transition rate is $r=8.4\times 10^{-4}$ and the weights in Equation (15) are $\phi_{2,2}^{\left(0\right)}=-0.437$, $\phi_{2,2}^{\left(1\right)}=0.351$, $\phi_{2,2}^{\left(2\right)}=0.539$ and $\phi_{2,2}^{\left(3\right)}=0.629$ (see Table 1). As expected, only the tripartite entangled state $|\phi_{2,2}\rangle$ becomes periodically populated during the evolution. The panel Figure 3e illustrates the behavior of the average excitations numbers of the qubits and the field in the presence of dissipation, together with the average total number of excitations $N_{tot}$ and $N_{tot}^{free}$ (under unitary evolution), confirming the feasibility of photon generation.

In Figure 4, we analyze the possibility of generation of several photons from vacuum due to the modulation-driven coupling between the states $|A_{0},n\rangle$ and $|A_{0},n+2\rangle$ near the degeneracy point $\Omega_{0}+\Omega_{a}\approx 2\nu$ ($n=0,2,4,\cdots ,n_{\max}$, where $n_{\max}$ denotes the most excited Fock state for a given modulation frequency). We assume that both qubits are far detuned from the cavity, so that $|A_{0},n\rangle \approx |g,g_{a},n\rangle$. Figure 4a shows the dimensionless transition rates $r_{n}=\left|R_{0,n;0,n+2}\right|/\nu$ for n=0,2,4 as function of $\Omega_{0}/\nu$, while Figure 4b show the resonant modulation frequencies $\eta_{n}=E_{0,n+2}-E_{0,n}$ for the same parameters as in Figure 2. The relative error of our analytic formulae is below 5% (0.1%) for the transition rates (modulation frequencies). One discontinuity in $\eta_{0}$ and two discontinuities in $\eta_{2}$ and $\eta_{4}$ are in agreement with the analytic results of Section 3.2, since the eigenvalues $E_{0,n\geq 2}$ present a single discontinuity near the degeneracy point (while $E_{0,0}$ is continuous). From these figures, we infer that at least three states $|A_{0},n\rangle$ with n=2,4,6 could be populated from the initial ground state provided the modulation frequency is sufficiently close to all the three frequencies $\eta_{0},\eta_{2}$ and $\eta_{4}$ (with the mismatch smaller or of the order of $\nu r_{n}$).

This hint is confirmed in Figure 4c, where we solved numerically the SE and the master equation for parameters $\Omega_{a}=0.6\nu$, $\Omega_{0}=1.405\nu$ and η=2.0086ν, when the transition rates are approximately $r_{0}=1.8\times 10^{-4}$, $r_{2}=1.1\times 10^{-4}$ and $r_{4}=9.6\times 10^{-5}$. This plot displays the average photon number, the average total excitation number and the Mandel’s Q-factor $Q=\langle {\left(\Delta \hat{n}\right)}^{2}\rangle /\langle \hat{n}\rangle -1$; bold (thin) lines depict the unitary (dissipative) evolution. Recall that *Q* quantifies the spread of the photon number distribution; some common values are Q=−1 (Fock state), Q=0 (coherent state), and $Q=\langle \hat{n}\rangle$ (thermal state). We see that a small number of photons can be created from vacuum even in the presence of dissipation, and the qubits remain approximately in the ground states because $N_{tot}$ is always close to $\langle \hat{n}\rangle$. The behavior of $\langle \hat{n}\rangle$ is better understood by looking at Figure 4d, in which we plot the photon number probabilities of the Fock states, $P_{n}=\mathrm{Tr}[\hat{\rho }|n\rangle \langle n|]$, with occupation probabilities above 10% under the unitary evolution. We see that up to six photons can be generated with significant probabilities, and the populations of the Fock states exhibit irregular oscillations due to the simultaneous coupling between the states $|A_{0},2n\rangle$ with n=0,…,3. The created field state is very different from the squeezed vacuum state generated in standard single-mode cavity DCE with vibrating walls or time-dependent permittivity [1], for which $Q=1+2\langle \hat{n}\rangle$. As expected, the dissipation destroys the oscillating behavior of $N_{tot}$, $\langle \hat{n}\rangle$, and *Q* after some time ($r_{0}\nu t≳\pi$ in Figure 4c) and these quantities tend to non-zero stationary values, loosing any resemblance with the unitary evolution.

### 4.1. Multi-Photon Transitions

For larger values of the cavity–ancilla coupling constant, g/ν∼ 0.2–0.3 (ultrastrong coupling regime [33,55]), generation of photons from vacuum becomes feasible via direct driving of the ground state of the Hamiltonian ${\hat{H}}_{0}$ to excited eigenstates with n>2 excitations. Here, we illustrate this rich variety of phenomena by considering the transitions from $|A_{0},0\rangle$ to the states $|A_{0},4\rangle$ and $|A_{0},6\rangle$. For the reasons already mentioned at the end of Section 3.2, it is more advantageous to obtain the transition rates and resonant modulation frequencies via numeric diagonalization of ${\hat{H}}_{0}$. To assess the experimental feasibility of these phenomena in open quantum systems we solve numerically the SMME, but warn that the obtained results serve only to attest that the corresponding transition rates are sufficiently high to overcome the dissipation for initial times. Although the quantitative (and even qualitative) accuracy of SMME cannot be guaranteed in the ultrastrong coupling regime, the obtained results are useful for indicating that during the time interval 0≤rνt≲π/2 the dissipative dynamics is qualitatively similar and not too different from the unitary one, so the generation of several photons should be possible.

In Figure 5a, we set the parameters $\Omega_{a}=0.6\nu$, g=0.2ν, and h=0.1ν, and evaluate numerically the dimensionless transition rate $r=|R_{0,0;0,4}|/\nu$ for the transition $|A_{0},0\rangle \to |A_{0},4\rangle$. As in previous figures, the transition rate presents sharp peaks near the degeneracies between $|A_{0},4\rangle$ and the states $|A_{2},1\rangle$ or $|A_{3},0\rangle$. The corresponding resonant modulation frequency $\eta_{r}=E_{0,4}-E_{0,0}$ is shown in Figure 5b. To confirm the feasibility of such multi-photon transition, we solved numerically the SE for $\Omega_{0}=3.12\nu$ and η=4.1873ν, when the transition rate is $r\approx 3.2\times 10^{-4}$ (hence larger than the dissipation rates). For these parameters, the ground state is $|A_{0},0\rangle \approx 0.99|A_{0}^{0}\rangle +0.13|A_{1}^{1}\rangle +0.02|A_{0}^{2}\rangle$ and the near degenerate dressed-states are $\left|0,4\rangle \approx 0.72\right|A_{0}^{4}\rangle -0.57|A_{1}^{3}\rangle -0.27|A_{2}^{1}\rangle +0.25|A_{1}^{5}\rangle +0.12|A_{3}^{0}\rangle$ (with approximate energy 4.1868ν above the ground state energy) and $\left|2,1\rangle \approx 0.86\right|A_{2}^{1}\rangle -0.39|A_{3}^{0}\rangle +0.22|A_{0}^{4}\rangle -0.18|A_{1}^{3}\rangle +0.16|A_{3}^{2}\rangle$ (with the corresponding energy 4.1899ν). Figure 5c shows the behavior of $\langle \hat{n}\rangle$, $N_{tot}$ and *Q* under unitary evolution, as well as the total excitation number $N_{tot}^{\left(dis\right)}$ assuming the standard dissipation kernel (6). We see that for rνt≲2, $N_{tot}$, and $N_{tot}^{\left(dis\right)}$ have similar behavior, confirming that the transition rate is sufficiently high to overcome the dissipation for initial times.

Figure 5d shows the excitation probability of t-qubit and ancilla for the initial zero-excitation state $|g,g_{a},0\rangle$, together with the fidelities $F_{k,l}={\left|\langle A_{k},l|\psi \left(t\right)\rangle \right|}^{2}$ during the unitary evolution. This intricate behavior occurs because the modulation couples the ground state $|A_{0},0\rangle$ to both the states $|A_{0},4\rangle$ and $|A_{2},1\rangle$, and we verified that $F_{0,0}+F_{0,4}+F_{2,1}>0.98$, proving that only the states $|A_{0},0\rangle$, $|A_{0},4\rangle$, and $|A_{2},1\rangle$ become significantly populated throughout the evolution. We see that nearly four excitations are created during the expected time interval rνt=π/2, and both qubits can be found in excited states with significant probabilities. The excitation of the ancilla comes mainly from the contribution of the state $|A_{1}^{3}\rangle$, and the excitation of the t-qubit comes mainly from the contribution of $|A_{2}^{1}\rangle$ in the dressed-states (since $|A_{1}\rangle \approx -|g,e_{a}\rangle$ and $|A_{2}\rangle \approx |e,g_{a}\rangle$ for the chosen parameters). Up to five photons can be detected with probabilities above 5%, and the contribution of the vacuum state becomes nearly zero for rνt≈π/2, as illustrated in Figure 5e.

Figure 6 is analogous to Figure 5 but for the direct transition $|A_{0},0\rangle \to |A_{0},6\rangle$. We performed numerical simulations for larger coupling strengths, g=0.3ν and h=0.2ν, but the same ancilla frequency $\Omega_{a}=0.6\nu$ as in Figure 4 and Figure 5. In panel Figure 6a, we verify a strong enhancement of the transition rate $r=|R_{0,0;0,6}|/\nu$ in the vicinity of degeneracy between $|A_{0},6\rangle$ and the states $|A_{2},1\rangle$ or $|A_{3},0\rangle$. The resonant modulation frequency $\eta_{r}=E_{0,6}-E_{0,0}$ is shown in Figure 6b. In panels Figure 6c–e, we solved numerically the SE for the initial state $|g,g_{a},0\rangle$ and parameters $\Omega_{0}=4.057\nu$ and η=5.201ν, when the transition rate is $r=4.6\times 10^{-4}$. The near degenerate states are $|A_{0},6\rangle \approx 0.68|A_{0}^{6}\rangle +0.52|A_{1}^{5}\rangle -0.39|A_{0}^{4}\rangle +0.25|A_{1}^{7}\rangle +0.16|A_{1}^{3}\rangle -0.14|A_{2}^{1}\rangle$ (with approximate energy 5.2025ν above the ground state energy) and $|A_{2},1\rangle \approx 0.8|A_{2}^{1}\rangle -0.52|A_{3}^{0}\rangle +0.25|A_{3}^{2}\rangle +0.11|A_{0}^{6}\rangle$ (with the corresponding energy 5.1978ν), while the ground state is $|A_{0},0\rangle \approx 0.98|A_{0}^{0}\rangle +0.19|A_{1}^{1}\rangle +0.04|A_{0}^{2}\rangle$. Figure 6c confirms the generation of photons with $\langle \hat{n}\rangle \leq 4.5$ and $N_{tot}\leq 5$ during the unitary evolution. Under dissipation, the plot of $N_{tot}^{\left(dis\right)}$ shows that for rνt≲1 the influence of dissipation is small, and generation of a few photons is feasible.The populations of Fock states with occupation probabilities above 10% are shown in Figure 6e (for unitary evolution). As expected, up to six photons are generated with significant probabilities (while the Fock states |2〉 and |3〉 are practically unpopulated), and the substantial population of the one-photon state comes from the partial excitation of the dressed-state $|A_{2},1\rangle$. Figure 6d shows the fidelities of the dressed states $|A_{0},6\rangle$, $|A_{2},1\rangle$ and $|A_{0},0\rangle$, whose sum is always above 96%, attesting that only these states become significantly populated during the evolution.

**Figure 7:**
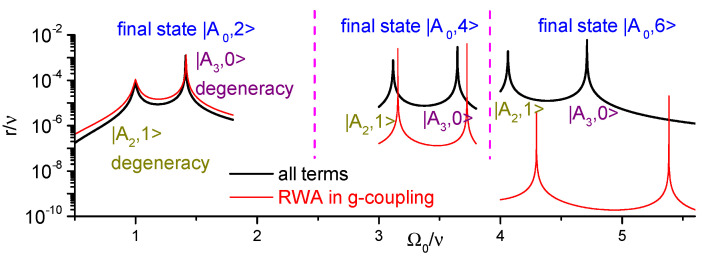
Comparison of the direct transition rates from the initial state $|A_{0},0\rangle$ to the states $|A_{0},2\rangle$, $|A_{0},4\rangle$, and $|A_{0},6\rangle$ (indicated on top) with the complete Hamiltonian ${\hat{H}}_{0}$ (black lines) and under RWA with $g(\hat{a}{\hat{\sigma }}_{-}^{\left(a\right)}+h.c.)=0$ (red lines). The parameters are the same as in Figure 4, Figure 5 and Figure 6, respectively. The peaks occur near degeneracies between the states {$|A_{0},n\rangle ,|A_{2},1\rangle$} and {$|A_{0},n\rangle ,|A_{3},0\rangle$} for n=2,4,6.

To conclude, we emphasize that in the regimes studied above the ancilla–cavity counter-rotating term $g(\hat{a}{\hat{\sigma }}_{-}^{\left(a\right)}+h.c.)$ in the Hamiltonian $\hat{H}$ plays an essential role. In the two-excitations transitions studied in Figure 2, Figure 3 and Figure 4, its major effect is to alter the resonant modulation frequencies and the position of peaks in transition rates, so in the first approximation it could be neglected via the Rotating Wave Approximation (RWA) (the transition rate would be wrong by roughly 30% in this case, provided one adjusted by hand the positions of the peaks). However, for 4- and 6-excitations transitions studied in Figure 5 and Figure 6 that term is indispensable, and the transition rates would be orders of magnitude smaller if it were neglected. This is illustrated in Figure 7, where we plot the transition rates of Figure 4a, Figure 5a and Figure 6a (corresponding to the direct transition $|A_{0},0\rangle \to |A_{0},n\rangle$, n=2,4,6) with and without the counter-rotating term and indicate the origin of each peak (degeneracy of $|A_{0},n\rangle$ with $|A_{2},1\rangle$ or $|A_{3},0\rangle$).

## 5. Conclusions

We showed that photons can be generated from a vacuum inside a stationary cavity due to the resonant time-modulation of a quantum system that is *indirectly coupled* to the field via some ancilla quantum subsystem. Such coupling may be advantageous when the modulated system is located outside or at the end of the resonator (when its coupling to the field is zero) to reduce the influence of the external driving on the cavity field, while it is strongly coupled to one or several ancilla subsystems. Moreover, this setup permits fabrication of quantum systems designed specifically for rapid external modulation at the cost of increased dissipation, while the stationary ancillas and cavities can be optimized for minimal dissipative losses.

We considered the simplest scenario of a single-mode cavity and a time-modulated qubit (we named “t-qubit”) playing the role of the indirectly coupled system, while the ancilla consisted of a stationary qubit dipole-coupled to both the cavity and the t-qubit. Our scheme and mathematical analysis can be readily generalized to more complex systems with alternative coupling mechanisms. We deduced analytically the system dynamics under unitary evolution and showed that the rate of photon generation can be drastically enhanced near certain resonances of the tripartite system (related to anti-crossings in the energy spectrum). We exemplified our scheme by studying photon generation from the initial vacuum state of the system, considering regimes in which either a single dressed-state or a small number of dressed-states with a few photons are populated under sinusoidal modulation. Moreover, we demonstrated that these phenomena can withstand dissipation with damping rates of the order of $10^{-5}$–$10^{-4}\nu$, since the typical photon generation rates are of the order of $10^{-4}\nu$ (where ν is the cavity frequency). Although the present study focused on prospects of photon creation from vacuum, our scheme could find applications in the engineering of effective interactions and generation of useful multipartite entangled states and non-classical states of light.

## Figures and Tables

**Table 1:** Probability amplitudes in the state $|\phi_{n=2,i=3}\rangle$, Equation (15), for $\Omega_{a}=\nu$ and $\Omega_{0}<\nu$.

t-Qubit Frequency	$\phi_{2,3}^{\left(0\right)}$	$\phi_{2,3}^{\left(1\right)}$	$\phi_{2,3}^{\left(2\right)}$	$\phi_{2,3}^{\left(3\right)}$
$\Omega_{0}/\nu =0.5$	0.735	−0.017	−0.678	0.012
$\Omega_{0}/\nu =0.7$	0.741	−0.048	−0.669	0.038
$\Omega_{0}/\nu =0.8$	0.740	−0.122	−0.653	0.105
$\Omega_{0}/\nu =0.85$	0.714	−0.240	−0.618	0.227
$\Omega_{0}/\nu =0.9$	0.579	−0.449	−0.479	0.484
$\Omega_{0}/\nu =0.95$	0.457	−0.538	−0.312	0.636
$\Omega_{0}/\nu =0.99$	0.489	−0.548	−0.178	0.655

**Table 2:** Probability amplitudes in the state $|\phi_{n=2,i=2}\rangle$ for $\Omega_{a}=\nu$ and $\Omega_{0}>\nu$.

t-Qubit Frequency	$\phi_{2,2}^{\left(0\right)}$	$\phi_{2,2}^{\left(1\right)}$	$\phi_{2,2}^{\left(2\right)}$	$\phi_{2,2}^{\left(3\right)}$
$\Omega_{0}/\nu =1.01$	−0.464	0.236	0.541	0.660
$\Omega_{0}/\nu =1.05$	−0.437	0.351	0.539	0.629
$\Omega_{0}/\nu =1.1$	−0.571	0.524	0.435	0.458
$\Omega_{0}/\nu =1.15$	−0.697	0.659	0.206	0.193
$\Omega_{0}/\nu =1.2$	−0.714	0.688	0.098	0.084
$\Omega_{0}/\nu =1.3$	−0.711	0.702	0.035	0.028
$\Omega_{0}/\nu =1.5$	−0.704	0.710	0.01	0.008

## Data Availability

The datasets used and/or analysed during the current study available from the corresponding author on reasonable request.

## Appendix A. Auxiliary Expressions

The eigenstates given in Equation (8) can be simplified. For $W_{+}\gg h$ the non-normalized states read approximately

(A1) $$\begin{array}{ccc} |A_{0}\rangle & \approx & |g,g_{a}\rangle -\left(h/2W_{+}\right)|e,e_{a}\rangle \\ |A_{3}\rangle & \approx & -|e,e_{a}\rangle -\left(h/2W_{+}\right)|g,g_{a}\rangle . \end{array}$$

For $\left|W_{-}\right|\ll h$ we have

(A2) $$\begin{array}{ccc} |A_{1}\rangle & \approx & |e,g_{a}\rangle -|g,e_{a}\rangle \\ |A_{2}\rangle & \approx & |e,g_{a}\rangle +|g,e_{a}\rangle , \end{array}$$

while for $\left|W_{-}\right|\gg h$

(A3) $$|A_{1}\rangle \approx \left\{\begin{array}{c} |e,g_{a}\rangle -(h/2|W_{-}|)|g,e_{a}\rangle \;,\;\Omega_{a}>\Omega_{0}\\ -|g,e_{a}\rangle +(h/2|W_{-}|)|e,g_{a}\rangle \;,\;\Omega_{a}<\Omega_{0} \end{array}\right.$$

(A4) $$|A_{2}\rangle \approx \left\{\begin{array}{c} |g,e_{a}\rangle +(h/2|W_{-}|)|e,g_{a}\rangle \;,\;\Omega_{a}>\Omega_{0}\\ |e,g_{a}\rangle +(h/2|W_{-}|)|g,e_{a}\rangle \;,\;\Omega_{a}<\Omega_{0}\;. \end{array}\right.$$

The coefficients of the state $|A_{0},n\rangle$ presented in Section 3.1 are

$$\Xi_{n}^{\left(1\right)}=\frac{1}{2v}\left(\frac{\sigma_{01}^{2}}{v-D_{+}+D_{-}}+\frac{\sigma_{02}^{2}}{v-D_{+}-D_{-}}\right)$$

$$\Xi_{n}^{\left(2\right)}=\frac{1}{2v}\left(\frac{\sigma_{01}^{2}}{v+D_{+}-D_{-}}+\frac{\sigma_{02}^{2}}{v+D_{+}+D_{-}}\right)$$

$$\begin{array}{ccc} \Xi_{n}^{\left(3\right)} & = & -\frac{\sigma_{01}\sigma_{02}D_{-}}{D_{+}}\left[\frac{n}{\left(v-D_{+}+D_{-}\right)\left(v-D_{+}-D_{-}\right)}\right.\\ & & +\left.\frac{n+1}{\left(v+D_{+}+D_{-}\right)\left(v+D_{+}-D_{-}\right)}\right] \end{array}$$

$$\Xi_{n}^{\left(4\right)}=\frac{\sigma_{01}\sigma_{02}D_{2}^{-}}{\left(v-D_{+}\right)\left(v-D_{+}-D_{-}\right)\left(v-D_{+}+D_{-}\right)}$$

$$\Xi_{n}^{\left(5\right)}=-\frac{\sigma_{01}\sigma_{02}D_{2}^{-}}{\left(v+D_{+}\right)\left(v+D_{+}+D_{-}\right)\left(v+D_{+}-D_{-}\right)}\;.$$

One also has a useful identity $\sigma_{01}^{2}+\sigma_{02}^{2}=1$.

The parameters of the matrix $M_{n}$, Equation (14), are: $a=g\sqrt{n}\sigma_{01}$, $b=g\sqrt{n}\sigma_{02}$, $c=g\sqrt{n-1}\sigma_{02}$, $d=g\sqrt{n-1}\sigma_{01}$, $x=D_{+}-D_{-}-\nu$, $y=D_{+}+D_{-}-\nu$ and $z=2(D_{+}-\nu )$. The eigenvalues $\Lambda_{n,i}$ of $M_{n}$, where the index *n* specifies the subspace and i=1,…,4 labels the eigenvalue, are the roots of the quartic equation

(A5) $$\Lambda_{n,i}^{4}+B\Lambda_{n,i}^{3}+C\Lambda_{n,i}^{2}+D\Lambda_{n,i}+E=0$$

with constant coefficients

(A6) $$\begin{array}{ccc} B & = & -\left(x+y+z\right)\\ C & = & xy+\left(x+y\right)z-a^{2}-b^{2}-c^{2}-d^{2}\\ D & = & \left(a^{2}+c^{2}\right)y+\left(b^{2}+d^{2}\right)x+\left(a^{2}+b^{2}\right)z-xyz\\ E & = & 2abcd+a^{2}d^{2}+b^{2}c^{2}-a^{2}yz-b^{2}xz\;. \end{array}$$

From the Ferrari’s method we obtain

(A7) $$\begin{array}{ccc} \Lambda_{n,1} & = & -\frac{B}{4}-S-\frac{1}{2}\sqrt{-4S^{2}-2p+\frac{q}{S}}\\ \Lambda_{n,2} & = & -\frac{B}{4}-S+\frac{1}{2}\sqrt{-4S^{2}-2p+\frac{q}{S}}\\ \Lambda_{n,3} & = & -\frac{B}{4}+S-\frac{1}{2}\sqrt{-4S^{2}-2p-\frac{q}{S}}\\ \Lambda_{n,4} & = & -\frac{B}{4}+S+\frac{1}{2}\sqrt{-4S^{2}-2p-\frac{q}{S}}\;, \end{array}$$

where

(A8) $$S=\sqrt{\frac{\Delta_{0}\cos\varphi -p}{6}}\;,\;q=D+\frac{B}{2}\left(\frac{B^{2}}{4}-C\right)$$

(A9) $$\varphi =\frac{1}{3}\arccos\left(\frac{\Delta_{1}}{2\Delta_{0}^{3}}\right)\;,\;p=C-\frac{3}{8}B^{2}$$

(A10) $$\Delta_{0}=\sqrt{C^{2}-3BD+12E}\;,\;\Delta_{1}=2C^{3}-9C\left(BD+8E\right)+27\left(B^{2}E+D^{2}\right)\;.$$

In Equation (15), the probability amplitudes $\phi_{n,i}^{\left(k\right)}$ of the conjoint atomic state $|A_{k}\rangle$ are

(A11) $$\phi_{n,i}^{\left(0\right)}=\frac{\Theta_{n,i}}{a}\left\{c-\frac{x-\Lambda_{n,i}}{c}\left[\left(z-\Lambda_{n,i}\right)+d\Phi_{n,i}\right]\right\}$$

$$\phi_{n,i}^{\left(1\right)}=\frac{\Theta_{n,i}}{c}\left[d\Phi_{n,i}+z-\Lambda_{n,i}\right]\;,\;\phi_{n,i}^{\left(2\right)}=\Theta_{n,i}\Phi_{n,i}\;,\;\phi_{n,i}^{\left(3\right)}=\Theta_{n,i}\;,$$

where

(A12) $$\Phi_{n,i}=\frac{\left[c-\left(x-\Lambda_{n,i}\right)\left(z-\Lambda_{n,i}\right)/c\right]\Lambda_{n,i}/a-a\left(z-\Lambda_{n,i}\right)/c}{b+\left[\Lambda_{n,i}\left(x-\Lambda_{n,i}\right)d/a+ad\right]/c}$$

(A13) $$\begin{array}{ccc} \Theta_{n,i} & = & \left\{1+\Phi_{n,i}^{2}+\frac{{\left[d\Phi_{n,i}+z-\Lambda_{n,i}\right]}^{2}}{c^{2}}\right.\\ & & +{\left.\frac{{\left[c^{2}-\left(x-\Lambda_{n,i}\right)\left(z-\Lambda_{n,i}\right)-\left(x-\Lambda_{n,i}\right)d\Phi_{n,i}\right]}^{2}}{a^{2}c^{2}}\right\}}^{-1/2}\;. \end{array}$$
